# Clinical efficacy and safety of a light mask for prevention of dark adaptation in treating and preventing progression of early diabetic macular oedema at 24 months (CLEOPATRA): a multicentre, phase 3, randomised controlled trial

**DOI:** 10.1016/S2213-8587(18)30036-6

**Published:** 2018-05

**Authors:** Sobha Sivaprasad, Joana C Vasconcelos, A Toby Prevost, Helen Holmes, Philip Hykin, Sheena George, Caroline Murphy, Joanna Kelly, Geoffrey B Arden, CLEOPATRA Study Group, Frank Ahfat, Ajay Bhatnagar, Nirodhini Narendran, Randhir Chavan, Abosede Cole, Roxanne Crosby-Nwaobi, Namritha Patrao, Deepthy Menon, Chris Hogg, Gary Rubin, Lauren Leitch-Devlin, Catherine Egan, Nisha Shah, Tatiana Mansour, Tunde Peto, Haralabos Eleftheriadis, Joanathan Gibson, Arevik Ghulakhszian, Gilli Vafidis, Edward Hughes, Afsar Jafree, Geeta Menon, Priya Prakash, Maria Sandinha, Richard Smith, Peter Scanlon, Steve Chave, Steve Aldington, Angela Dale, Gillian Hood, Graham A Hitman, David Crabb, Alaistair Denniston, Douglas Lewin, Ian Grierson, Sarah Walker, Jackie Sturt, Debendra Sahu

**Affiliations:** a National Institute for Health Research (NIHR) Biomedical Research Centre at Moorfields Eye Hospital and UCL Institute of Ophthalmology, London, UK; b Imperial Clinical Trials Unit, School of Public Health, Imperial College London, London, UK; c King's Clinical Trials Unit at King's Health Partners, King's College London, London, UK; d Hillingdon Hospital, Hillingdon Hospitals National Health Service Foundation Trust, Uxbridge, UK; e Institute of Ophthalmology and Moorfields Eye Hospital, London, UK

## Abstract

**Background:**

We aimed to assess 24-month outcomes of wearing an organic light-emitting sleep mask as an intervention to treat and prevent progression of non-central diabetic macular oedema.

**Methods:**

CLEOPATRA was a phase 3, single-blind, parallel-group, randomised controlled trial undertaken at 15 ophthalmic centres in the UK. Adults with non-centre-involving diabetic macular oedema were randomly assigned (1:1) to wearing either a light mask during sleep (Noctura 400 Sleep Mask, PolyPhotonix Medical, Sedgefield, UK) or a sham (non-light) mask, for 24 months. Randomisation was by minimisation generated by a central web-based computer system. Outcome assessors were masked technicians and optometrists. The primary outcome was the change in maximum retinal thickness on optical coherence tomography (OCT) at 24 months, analysed using a linear mixed-effects model incorporating 4-monthly measurements and baseline adjustment. Analysis was done using the intention-to-treat principle in all randomised patients with OCT data. Safety was assessed in all patients. This trial is registered with Controlled-Trials.com, number ISRCTN85596558.

**Findings:**

Between April 10, 2014, and June 15, 2015, 308 patients were randomly assigned to wearing the light mask (n=155) or a sham mask (n=153). 277 patients (144 assigned the light mask and 133 the sham mask) contributed to the mixed-effects model over time, including 246 patients with OCT data at 24 months. The change in maximum retinal thickness at 24 months did not differ between treatment groups (mean change −9·2 μm [SE 2·5] for the light mask *vs* −12·9 μm [SE 2·9] for the sham mask; adjusted mean difference −0·65 μm, 95% CI −6·90 to 5·59; p=0·84). Median compliance with wearing the light mask at 24 months was 19·5% (IQR 1·9–51·6). No serious adverse events were related to either mask. The most frequent adverse events related to the assigned treatment were discomfort on the eyes (14 with the light mask *vs* seven with the sham mask), painful, sticky, or watery eyes (14 *vs* six), and sleep disturbance (seven *vs* one).

**Interpretation:**

The light mask as used in this study did not confer long-term therapeutic benefit on non-centre-involving diabetic macular oedema and the study does not support its use for this indication.

**Funding:**

The Efficacy and Mechanism Evaluation Programme, a Medical Research Council and National Institute for Health Research partnership.

## Introduction

**Figure 1:**
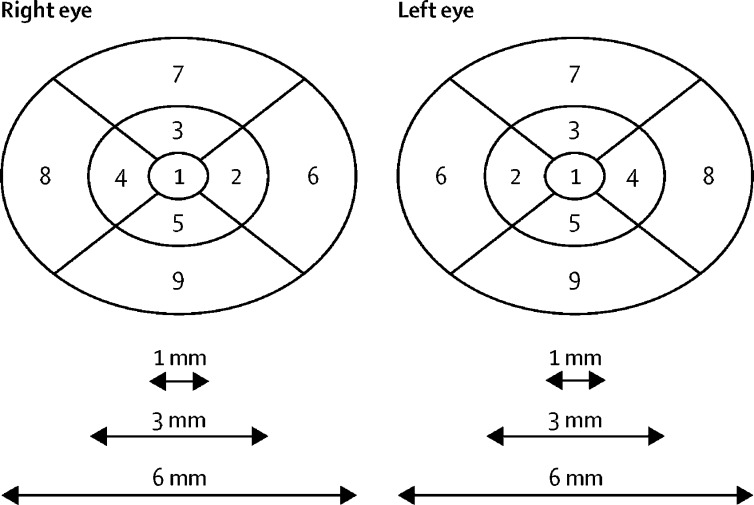
ETDRS grid within OCT devices The ETDRS grid divides the macula into nine zones in the right and left eye, with zone 1 the central subfield, zones 2–5 parafoveal zones, and zones 6–9 perifoveal zones. ETDRS=Early Treatment Diabetic Retinopathy Study. OCT=optical coherence tomography.

Diabetic macular oedema is the most common cause of moderate visual loss in people with diabetes mellitus.^1^ The location and quantity of increased retinal thickness due to diabetic macular oedema can be recorded objectively using optical coherence tomography (OCT). All OCT devices have an in-built Early Treatment Diabetic Retinopathy Study (ETDRS) grid with nine zones (subfields) centred on the fovea (figure 1). Centre-involving diabetic macular oedema is defined as presence of oedema in zone 1. Non-centre-involving diabetic macular oedema is oedema restricted to one or more of zones 2–9. The zone of maximum retinal thickness can be monitored over time to assess the course of disease. However, diabetic macular oedema can appear and disappear at any zone and might represent worsening of disease despite resolution of oedema at the zone of maximum retinal thickness. Therefore, response to treatment must be assessed objectively by looking at several variables, including change in retinal thickness in all nine zones as well as total macular volume.^2^ Not all patients with centre-involving diabetic macular oedema have impaired vision. Therefore, progression of diabetic macular oedema to the centre is better measured as an increase in central subfield thickness (zone 1) than change in visual acuity.

Approximately 8% of people with diabetes have centre-involving diabetic macular oedema and a further 8% have non-central diabetic macular oedema. Standard treatments for people with centre-involving diabetic macular oedema are invasive and include repeated intravitreal injections of anti-vascular endothelial growth factor (anti-VEGF) agents, use of steroids, or laser treatment.^3^, ^4^ Less invasive treatment will save patients from these procedures and have a major positive effect on health-care spending. Furthermore, there is an unmet need for preventive and treatment options for non-central diabetic macular oedema to prevent potential visual morbidity due to disease progression.^5^

**Research in context**

**Evidence before this study**

We searched PubMed from database inception until Sept 1, 2017, and abstracts from annual meetings of the Association for Research in Vision and Ophthalmology until 2017, with the terms “lightmasks” AND “diabetic retinopathy” OR “diabetic macular oedema” for reports of randomised controlled trials published in English only. We identified no randomised trials. Non-centre-involving diabetic macular oedema can progress to the centre of the macula and cause visual impairment. Standard treatment for centre-involving diabetic macular oedema with visual impairment is repeated intravitreal injections with anti-vascular endothelial growth factor agents. Other treatment options include macular laser and intravitreal steroids. Many investigators have evaluated less invasive treatment options for non-centre-involving macular oedema to delay or prevent disease progression but have been unsuccessful. Rod photoreceptors in the retina consume a maximum amount of oxygen during dark adaptation. In diabetes, the resultant hypoxia can contribute to development and progression of diabetic macular oedema and diabetic retinopathy. Two short-term clinical trials showed that wearing light masks emitting 500–505 nm light through the eyelids to decrease dark adaptation reduced the rate of progression of diabetic retinopathy and early diabetic macular oedema, respectively. In an abstract, 45 healthy volunteers wore an organic light-emitting sleep mask to prevent dark adaptation and showed no safety concerns at 4 months. 24% withdrew from the intervention before 1 month because of light intolerance and sleep disturbance. In another abstract, a light-emitting sleep mask to prevent dark adaptation in six patients with refractory clinically significant diabetic macular oedema showed good acceptability and tolerance for five patients at 6 months. As far as we know, no randomised controlled trials have been done to assess the role of light masks during sleep as a novel treatment for patients with non-central diabetic macular oedema. A study assessing the safety and acceptability of an organic light-emitting sleep mask (Noctura 400 Sleep Mask, PolyPhotonix Medical, Sedgefield, UK) in healthy volunteers (n=45) and patients with diabetic macular oedema (n=15) reported no clinically relevant safety issues at 4 months. 16 withdrew from the study, eight before month 1. The mean change in maximum retinal thickness in eyes with diabetic macular oedema at 4 months was −12·00 μm (range −28·80 to 4·80). A recent publication also showed good compliance with the light masks in diabetic macular oedema at 6 months.

**Added value of this study**

The CLEOPATRA trial is, to our knowledge, the first randomised controlled trial to evaluate the effect of an organic light-emitting sleep mask as a treatment option for non-centre-involving diabetic macular oedema. The 24-month follow-up period provides data for efficacy, safety, and compliance of wearing these light masks for this condition. The results show that the light masks as used in this study did not provide any discernible clinical benefit. No differences were recorded between wearing and not wearing these light masks in the change in thickness in the zone of maximum retinal thickness, total macular volume, progression of the oedema to the centre, proportion of patients requiring standard treatment for diabetic macular oedema, and progression of diabetic retinopathy. The analysis of compliance highlighted that wearing these light masks over 24 months might also not be a sustainable option, as compliance decreased over time. The results of the study were not accounted for by non-compliance of wearing the light masks. No light mask-related serious adverse events were recorded.

**Implications of all the available evidence**

The CLEOPATRA study provides evidence that the light mask offered to prevent dark adaptation is not recommended as a treatment option for non-centre-involving diabetic macular oedema. Although earlier studies showed short-term improvement in diabetic oedema and diabetic retinopathy using 505 nm light masks, our study shows that compliance wearing the light masks during sleep is challenging and is therefore not a sustainable option. Since laboratory-based evidence of the role of photoreceptors in diabetic retinopathy is increasing, there remains an unmet need to translate this idea into interventions in patients.

During dark adaptation, normal rod photoreceptors in the retina consume nearly all the oxygen available to the eye.^6^ In patients with diabetes the retinal oxygen supply is compromised and the hypoxic status during dark periods might exacerbate microvascular changes.^7^ This idea has been substantiated by the fact that oxygen supplementation alleviates diabetic macular oedema in the short term.^8^ Sivaprasad and Arden^7^ postulated that if dark adaptation could be prevented, diabetic macular oedema and diabetic retinopathy might be alleviated by decreasing the oxygen demand. Since dark adaptation in man only happens at night during sleep, sleeping in an environment illuminated with 500–505 nm light should suppress rods and prevent or reverse diabetic macular oedema. A proof-of-concept study in 12 patients who slept at night using a mask containing a chemiluminescent source that exposed one eye only to light for 3 months showed that the treatment had no safety issues, was acceptable to patients, and both colour vision and microaneurysm count improved.^9^ A second study used light-emitting diodes (LEDs) to illuminate one eye with 505 nm light during sleep in 40 patients with bilateral diabetic macular oedema.^10^ 34 patients completed the study and an improvement in retinal function and a decrease in retinal thickness at 6 months was noted. Based on these observations, the Noctura 400 Sleep Mask (PolyPhotonix Medical, Sedgefield, UK) was CE-approved for the treatment of diabetic retinopathy. The long-term effectiveness, compliance, and safety of light masks are unknown. We did a phase 3 clinical trial (CLEOPATRA) to investigate whether offering the light mask to wear over closed eyelids during sleep at night for 24 months could treat and prevent the progression of non-centre-involving diabetic macular oedema.

## Methods

### Study design and patients

The CLEOPATRA study is a phase 3, multicentre, single-blind, parallel-group, randomised controlled trial. Patients were recruited from 15 ophthalmic centres at UK National Health Service (NHS) hospitals. We included adults (aged ≥18 years) with type 1 or 2 diabetes mellitus and clinical and OCT evidence of retinal thickening in at least one non-central ETDRS zone due to diabetic macular oedema with best-corrected visual acuity of more than 55 ETDRS letters, equivalent to 6/18 Snellen. We permitted previous macular laser therapy, intravitreal steroids, or anti-VEGF agents provided the last treatment was given at least 4 months before randomisation.^11^

Exclusion criteria for eyes were centre-involving diabetic macular oedema, other causes of macular oedema, or coexistent ocular disease that affected or might affect visual acuity or prevent treatment delivery. We also excluded eyes with active proliferative diabetic retinopathy or that were treated previously with panretinal photocoagulation. Systemic exclusion criteria included history of insomnia or any other sleep disturbances.

The study was granted approval by the National Research Ethics Committee Service London—Dulwich (13/LO/0145). Trial Steering and Data Monitoring Committees provided independent oversight. A representative of the manufacturer was a non-voting member of the Trial Steering Committee. All eligible patients gave written informed consent before study participation.

### Randomisation and masking

We randomly allocated eligible patients (1:1) to wear during sleep either a light mask or a sham (non-light) mask, using the method of minimisation, concealed before allocation, stratified by HbA_1c_ (<8% [63·89 mmol/mol] or ≥8% [63·90 mmol/mol]), perifoveal (ETDRS zones 6–9) versus parafoveal (ETDRS zones 2–5) baseline thickness in excess of 320 μm in the perifoveal or parafoveal zones, and study site. For patients with the same baseline thickness in excess of 320 μm in the perifoveal or parafoveal zones, the parafoveal zone was chosen. Randomisation was done by collaborating site investigators via the King's Clinical Trials Unit web-based randomisation service. Patients and examining clinicians were aware of the study allocation because of the nature of the intervention. Patients assigned the sham mask had the option of not using it because it became apparent early in the study that many patients were not using it. Outcome assessors including OCT technicians, optometrists, and graders at the independent reading centre based at the Gloucestershire Eye Unit (Gloucester, UK) were unaware of treatment allocation.

### Procedures

The light mask used in the intervention arm was the Noctura 400 Sleep Mask (PolyPhotonix Medical). This CE-certified class 2a device is designed to deliver blue-green light through closed eyelids. The light mask consists of two battery-operated organic LEDs inserted within a fabric mask and placed over the patients' eyes using an adjustable velcro strap. It is operational for a maximum of 8 h therapy per night. The lifetime of the light mask is 84 days, after which time a replacement mask is required. Based on calculations done by the manufacturer, the light mask provides a luminance of 75 photopic cd/m^2^ (± 10%), equating to 186 scotopic cd/m^2^. After considering light attenuation through closed eyelids and pupillary diameter, these light masks are expected to cause 40% reduction in rod-circulating current. The decay of mask output over its lifetime is also maintained within 10% of the desired output. The light intensity we used is approximately six orders of magnitude less than for threshold toxicity and two orders below that which causes a 1% change in the melatonin cycle that drives circadian rhythms.

The light mask records automatically when it is being worn, providing an accurate measure of compliance. These data were downloaded by study sites when masks were returned. The manufacturer was also sent anonymised data from every returned light mask to measure compliance. We took the pragmatic decision that 6 h/day (4380 h over 2 years) was sufficient to represent 100% compliance and, therefore, represented the level at and above which maximum benefit would be derived. We defined compliance as patients who wore the masks 70% of the time (3066 h, counting time truncated to 6 h/day). If compliance data were missing for a day then we assumed no compliance (ie, the mask had not been worn that night).

The trial manager contacted study sites to request they take steps to maximise the rate of mask return, to ensure availability of compliance data. The trial manager followed the trial monitoring plan by undertaking off-site monitoring on a monthly basis. This process included contacting sites at which patients' compliance was less than 40%. The manufacturer also alerted the trial manager when patients had poor compliance. Moreover, during every on-site monitoring visit, sites were asked to reinforce with patients the importance of wearing the masks every night during sleep, and specific patients with issues of compliance were discussed. The Data Monitoring Committee, in closed meetings, reviewed the accumulating compliance data and concluded that a dose-effect of the light masks should also be evaluated by comparing the effect of the light masks at three levels of compliance (50%, 60%, and 70%). The protocol was amended to this effect and approved by the Trial Steering Committee, the sponsor, and the Research Ethics Committee.

The clinical assessments schedule is detailed in the appendix (p 1) and in the published protocol.^11^ We recorded HbA_1c_ at baseline, 12 months, and 24 months. Patients had OCT assessments every 4 months, and these assessments were done twice at 12 months and 24 months to ensure that the treatment effect was distinguished from the test-retest variability. We recorded concomitant diabetic medications, anti-VEGF agents, steroids, and laser treatment throughout the study. We measured refracted best-corrected visual acuity at baseline, 12 months, and 24 months using validated ETDRS visual acuity charts, and we repeated these measurements at baseline to assess test-retest variability. We did three-field colour fundus photography at baseline, 12 months, and 24 months to grade the severity of diabetic retinopathy. Both the examining clinician and graders at the independent reading centre graded anatomical characteristics of the diabetic macular oedema and severity of diabetic retinopathy. We defined an improvement in severity score for diabetic retinopathy as the proportion of patients with an ETDRS severity level of 2 or higher, at 12 months and 24 months (appendix pp 2, 3). We measured sleep disturbances at 12 months and 24 months. We used the Epworth Sleepiness Scale (ESS) to assess changes in daytime sleepiness, with scores ranging from 0 (low level of daytime sleepiness) to 24 (high level of daytime sleepiness),^12^ and the Pittsburgh Insomnia Rating Scale—20 item version (PIRS-20) to assess changes in insomnia, with scores ranging from 0 (no insomnia) to 60 (worse insomnia).^13^

We recorded adverse events at every visit. We analysed differences from baseline to 24 months in ocular and systemic safety profiles with the light mask relative to the sham mask. Two clinicians who were unaware of treatment allocation coded ocular and systemic adverse events.

A subset of patients (n=30) also underwent oximetry, multifocal electroretinography, and microperimetry before and after 100% oxygen at baseline and 12 months. This mechanistic component of the study will be reported later.

### Outcomes

The primary outcome was the change from baseline to 24 months in maximum retinal thickness in the study eye with the light mask relative to the sham mask, measured by OCT. For participants with the same maximum baseline retinal thickness in two zones, the zone located in the parafoveal zone was chosen. When these two zones were in the parafoveal zone, the average retinal thickness was taken in subsequent follow-up measurements. For 12-month and 24-month measurements, OCT was done twice and the average of the measurements was taken.

A per-protocol secondary analysis excluded data from the point at which any patient was treated for worsening diabetic macular oedema. Additional secondary outcomes assessed at 12 months and 24 months included changes in thickness in the central subfield zone, zones 1–5, and zones 1–9, total macular volume, and morphological characteristics of diabetic macular oedema. We also assessed the change in refracted best-corrected visual acuity from baseline at 12 months and 24 months. Disease progression outcomes included time to occurrence of centre-involving diabetic macular oedema (defined as >300 μm), the proportion of patients progressing to centre-involving diabetic macular oedema of 400 μm or greater (ie, they met eligibility criteria for treatment with anti-VEGF agents in England and Wales), and the number of patients who received standard treatment for diabetic macular oedema at 12 months and 24 months (including anti-VEGF agents, steroids, and macular laser therapy).

### Statistical analysis

The pilot for this intervention^8^ provided an SD for the change from baseline in retinal thickness of 35·68 μm and informed 20% attrition. The detectable effect size of 15 μm was plausible relative to the 95% CI, and was minimally distinguishable from the 10·2 μm test–retest variation, for which the test–retest mean change over time of 0·9 μm was adequately small. A sample size of 300 patients (150 per treatment group)—with 240 patients analysed—provided 90% power based on a two-sided, unpaired *t* test at the 5% level of significance. Standardised effect sizes of 0·42 between treatment groups were detectable for secondary outcomes—eg, change in visual acuity. The planned statistical analysis incorporated serial measures and baseline adjustment, ensuring an improvement in power and in the precision of estimated treatment effects on each outcome.

We finalised the statistical analysis plan before data lock and agreed it with the oversight committees (Trial Steering and Data Monitoring Committees). We analysed the primary outcome with a linear mixed-effects model, incorporating six 4-monthly post-baseline observations of the outcome over time to 24 months and accommodating the within-participant correlation over time with an unstructured covariance matrix.^14^ The model included fixed factors for treatment group, HbA_1c_, and study site, and the continuous baseline of the outcome, each interacting with time. We did a sensitivity assessment of the missing-at-random assumption made in the primary outcome analysis in all patients, with three recommended scenarios affecting either one or both treatment groups, making this an intention-to-treat strategy,^15^, ^16^ with the intention-to-treat population comprising all randomised patients with OCT data. We did a per-protocol secondary analysis in which we excluded data from randomised patients at the point at which they were treated with steroids, anti-VEGF agents, or laser therapy, because these treatments could substantially improve retinal thickness after deterioration. We analysed secondary continuous outcomes with the same model specification as for the primary outcome, and with a missing baseline indicator if needed,^17^ and we reported data as adjusted differences in means. All tests were two-sided at the 5% significance level and effect sizes were interpreted cautiously with 95% CIs. We used the *t* test to compare means, the χ^2^ test or Fisher's exact test for single proportions, McNemar's SE for changes in proportions, and the Kaplan-Meier test for cumulative proportions.^18^

We used complier average causal effect (CACE) analysis to estimate efficacy in patients who complied with treatment. We defined compliance, in turn, as wearing the assigned mask 70%, 60%, and 50% of the time, assuming the missing-at-random assumption in the primary outcome model and no effect of randomisation on outcome in non-compliers.^19^

We did sensitivity analyses of patients who met the requirement for treatment of centre-involving diabetic macular oedema because the central subfield thickness reached 400 μm before the 24-month endpoint, on the time to reaching 400 μm, and on the potential differential variability in retinal thickness between treatment groups (with the Mann-Whitney test). Because Spectralis (Heidelberg Engineering, Heidelberg, Germany) is the only OCT device with automatic real-time tracking, we did a sensitivity analysis including only patients who had OCT outcomes captured with this device at baseline. We also did a sensitivity analysis to exclude outliers defined as 4 SD from expected, which we do not present here because no changes were recorded in primary or secondary outcome analysis conclusions. We used IBM SPSS Statistics version 23 for statistical analyses.

This trial is registered with Controlled-Trials.com, number ISRCTN85596558.

### Role of the funding source

The funder had no role in study design, data collection, data analysis, data interpretation, or writing of the report. The manufacturer of the light mask provided input into protocol development and trained site staff to offer the light mask as per protocol and their instructions for use manual. The manufacturers were sent anonymised data from every returned light mask for measurement of compliance. They provided feedback to the study team on masks that showed low compliance so clinical site staff could be informed and asked to reinforce use. The statisticians (ATP and JCV) had full access to all data in the study and the chief investigator (SS) had final responsibility for the decision to submit the results for publication.

## Results

**Figure 2:**
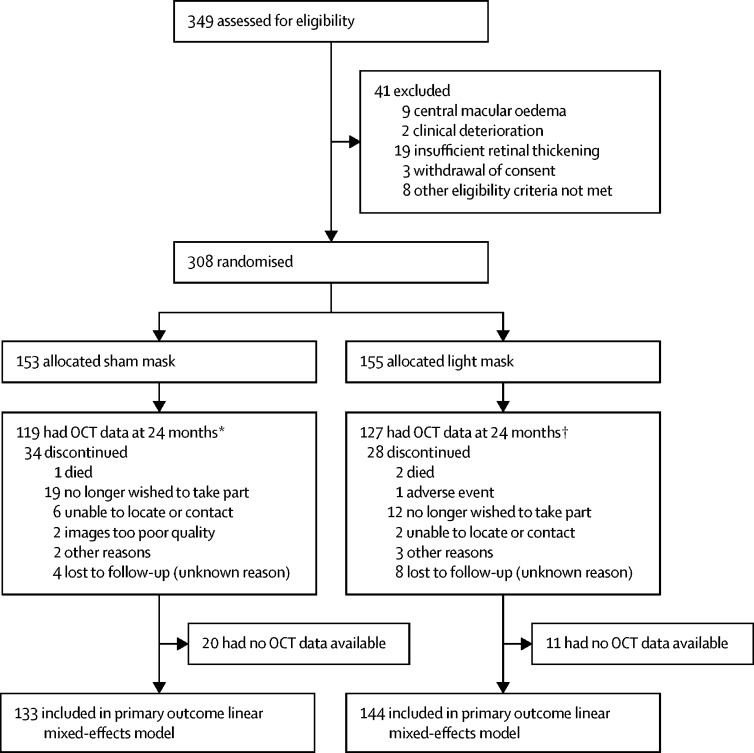
Trial profile OCT=optical coherence tomography. *Includes four patients lost to follow-up who had clinical OCT data. †Includes five patients lost to follow-up who had clinical OCT data.

Between April 10, 2014, and June 15, 2015, 349 patients were assessed for eligibility. 41 patients did not meet eligibility criteria and were excluded; thus, 308 participants were randomly assigned to receive either the light mask (n=155) or the sham mask (n=153; figure 2).

Baseline characteristics were well balanced between treatment groups (table 1). The mean age of patients was 57 years (SD 11). 194 (63%) of 308 participants were men. 286 (93%) had baseline maximum retinal thickness in parafoveal zones 2–5 whereas 22 (7%) had maximum retinal thickness in perifoveal zones 6–9. 154 (50%) patients had HbA_1c_ less than 8% (63·89 mmol/mol) at baseline. The average of the two refracted visual acuity measurements at baseline was mean 84·3 (SD 7·3) ETDRS letters, which was equivalent to 6/6 Snellen. 183 (59%) of 308 patients had OCT measurements taken with Spectralis (Heidelberg Engineering), 54 (18%) with Cirrus (Carl Zeiss Meditec, Cambridge, UK), 61 (20%) with Topcon 2000 (Topcon, Tokyo, Japan), and ten (3%) with RS3000 (Nidek, Aichi, Japan). 62 patients did not have primary outcome data (figure 2); no differences in baseline characteristics were noted between patients who dropped out and those who did not, except for study site, which was already adjusted for in the analysis (appendix p 4). This finding was attributable largely to one study site having a high dropout rate.

**Table 1:** Baseline characteristics

		**Sham mask (n=153)**	**Light mask (n=155)**
Age (years)		59·0 (51·0–67·0)	57·0 (51·0–65·0)
Sex			
	Men	92 (60%)	102 (66%)
	Women	61 (40%)	53 (34%)
Ethnic origin			
	White	94 (61%)	100 (65%)
	Black	29 (19%)	27 (17%)
	Asian	28 (18%)	24 (15%)
	Other	2 (1%)	4 (3%)
Smoker		10 (7%)	13 (8%)
Study site			
	Bristol Eye Hospital	6 (4%)	6 (4%)
	Birmingham Heartlands Hospital	4 (3%)	7 (5%)
	Sandwell & West Birmingham Hospitals NHS Trust	3 (2%)	3 (2%)
	Frimley Park Hospital	16 (10%)	16 (10%)
	Hillingdon Hospital	27 (18%)	27 (17%)
	King's College Hospital	17 (11%)	18 (12%)
	Moorfields Eye Hospital	25 (16%)	26 (17%)
	Central Middlesex Hospital	3 (2%)	1 (1%)
	Maidstone & Tunbridge Wells Hospital	6 (4%)	6 (4%)
	Princess Alexandra Hospital, Harlow	6 (4%)	5 (3%)
	The Royal Wolverhampton NHS Trust	11 (7%)	10 (6%)
	Brighton & Sussex University Hospitals NHS Trust	1 (1%)	2 (1%)
	Sunderland Eye Infirmary	13 (8%)	12 (8%)
	Stoke Mandeville Hospital	5 (3%)	5 (3%)
	William Harvey Hospital Kent	10 (7%)	11 (7%)
Blood pressure (mm Hg)			
	Systolic	140·3 (18·9)*	137·2 (16·5)*
	Diastolic	81·0 (10·2)*	80·2 (9·4)*
Diabetes mellitus			
	Type 1	20 (13%)	31 (20%)
	Type 2	133 (87%)	124 (80%)
Medication			
	Insulin only	22 (14%)	43 (28%)
	Oral hypoglycaemic agents only	75 (49%)	72 (46%)
	Insulin and oral hypoglycaemic agents	56 (37%)	39 (25%)
	Diet-controlled	0	1 (1%)
Best-corrected visual acuity (ETDRS letters)		86·0 (81·3–89·0)	85·5 (81·5–89·0)
Maximum retinal thickness (μm)		348·8 (24·3)	345·9 (21·6)
Total volume (mm^3^)		8·7 (8·3–9·3)	8·7 (8·3–9·2)
HbA_1c_			
	<8% (<63·89 mmol/mol)	77 (50%)	77 (50%)
	≥8% (≥63·90 mmol/mol)	76 (50%)	78 (50%)
Severity level (study eye)†			
	10	2 (1%)	2 (1%)
	20	25 (17%)	35 (23%)
	35	101 (69%)	93 (60%)
	43–47	11 (7%)	15 (10%)
	53	0	1 (1%)
	61‡	0	2 (1%)
	65‡	1 (1%)	1 (1%)
	71–75‡	0	1 (1%)
	81–85‡	0	0
	90‡	7 (5%)	4 (3%)
Intraocular pressure (mm Hg)		16·0 (14·0–18·0)*	16·0 (14·0–18·0)§

Data are median (IQR), mean (SD), or number of participants (%). ETDRS=Early Treatment Diabetic Retinopathy Study.

* Data missing for one participant.

† Data from the independent reading centre; data missing for five participants assigned the sham mask and one allocated the light mask.

‡ Participants with these severity levels should have been excluded.

§ Data missing for two participants.

For the prespecified primary outcome analysis, OCT data were available for 246 (80%) of 308 patients at 24 months, of whom 127 were assigned the light mask and 119 were allocated the sham mask. This number includes five patients assigned the light mask and four patients allocated the sham mask, for whom OCT data were obtained from routine clinical care (ie, the patient attended their clinic appointment but did not attend an intervening research visit). An additional 17 patients assigned the light mask and 14 allocated the sham mask had OCT data from previous timepoints (appendix p 5). Therefore, 277 (90%) of 308 patients were included in the intention-to-treat linear mixed-effects model, of whom 144 had been assigned the light mask and 133 had been allocated the sham mask. At 24 months, in the 246 patients who had data available, no difference was recorded in mean change in maximum retinal thickness between the light mask and sham mask (adjusted difference −0·65 μm, 95% CI −6·90 to 5·59; p=0·84; table 2). Furthermore, no difference was noted in mean change in maximum retinal thickness between treatment groups at any timepoint (appendix p 6).

**Table 2:** Maximum retinal thickness measured by OCT at baseline, 12 months, and 24 months

	**Maximum retinal thickness (μm)**		**Mean (SE) change from baseline (μm)**		Adjusted difference (95% CI)*(μm)	**p value**
	Sham mask	Light mask	Sham mask	Light mask		
Baseline†	348·8 (24·3), n=153	345·9 (21·6), n=155	..	..	..	..
12 months	339·1 (35·9), n=121	341·3 (29·7), n=132	−9·5 (3·1)	−4·6 (2·5)	1·73 (−5·31 to 8·77)	0·63
24 months	336·3 (29·7), n=119	336·0 (25·5), n=127	−12·9 (2·9)	−9·2 (2·5)	−0·65 (−6·90 to 5·59)	0·84

Data are mean (SD), number of participants, unless otherwise indicated. 277 patients were included in the linear-mixed effects model. OCT=optical coherence tomography.

* Adjusted for HbA_1c_, study site, and baseline maximum retinal thickness.

† Mean maximum baseline retinal thickness for 133 patients assigned the sham mask and included in the linear mixed-effects model was 348·6 μm (SD 24·2) and for 144 patients allocated the light mask it was 345·4 μm (21·2). Mean maximum baseline retinal thickness for 20 patients assigned the sham mask and not included in the model was 350·6 μm (SD 25·6) and for 11 patients allocated the light mask it was 352·9 μm (26·6).

For the per-protocol secondary analysis of change in maximum retinal thickness, in which patients were excluded at the point they began treatment for diabetic macular oedema (laser therapy, steroids, or anti-VEGF agents; appendix p 7), 266 patients were included in the linear mixed-effects model at 12 months and 24 months (the linear mixed-effects model takes into account data at all timepoints). 56 patients needed treatment for diabetic macular oedema, of whom 23 had been assigned the light mask and 33 had been allocated the sham mask. The difference in the cumulative proportion of patients requiring treatment between treatment groups was 8% (95% CI 0–16) at 12 months and 9% (1–18) at 24 months. The change in maximum retinal thickness did not differ between the light mask and sham mask at 12 months and 24 months (adjusted difference at 24 months 3·23 μm, 95% CI −2·11 to 8·58; p=0·23; appendix p 8).

Median compliance with wearing the light mask was 39·5% (IQR 9·8–78·2) at 4 months, falling to 19·5% (1·9–51·6) at 24 months. When considering the three definitions of compliance (ie, 70%, 60%, and 50%, with 70% compliance meaning the light mask was used 70% of the available time in the study, up to 6 h/day counted daily), the proportions of patients achieving each of these three levels of compliance decreased over time (appendix p 9).

Sensitivity analyses for missing data were done to represent three possible scenarios, to reflect whether departures from the missing-at-random assumption applied within patients assigned the light mask only, within those allocated the sham mask only, and within both treatment groups equally and in the same direction (appendix p 10). The change in maximum retinal thickness did not differ between use of the light mask and the sham mask for all three scenarios. Assuming patients with unobserved outcome data in one or both treatment groups would take values as much as a prespecified 20 μm either side of the adjusted observed effect, all 95% CIs included 0 and excluded −15, thus confirming that the absence of a clinically important light mask effect is robust to missing data (appendix p 11). In the sensitivity analysis for non-compliance, the CACE estimate for compliers defined by 70% compliance was −4·2 (95% CI −44·6 to 36·1), 60% compliance was −3·1 (−32·4 to 26·3), and 50% compliance was −2·5 (−26·7 to 21·7). Across these three definitions of compliers, the results were consistent in estimating a small non-significant intervention effect, which was not close to the detectable effect of 15 μm retinal thickness.

In the sensitivity analysis of patients who met the requirement for treatment of centre-involving diabetic macular oedema (ie, retinal thickness reached 400 μm before the 24-month endpoint), the retinal thickness measurement taken just after the participant first reached 400 μm was carried forward to be their final measurement. Nine patients achieved 400 μm in central macula, of whom three had been assigned the sham mask (all achieved this point at 24 months) and six had been allocated the light mask (two achieved this point at 24 months, two at 20 months, and two at 12 months). 161 patients were included in this linear mixed-effects model and the adjusted difference between treatment groups was −0·22 μm (95% CI −8·36 to 7·92; p=0·96). 259 patients were included in the Cox proportional hazards regression time-to-event analysis (ie, time to reaching 400 μm), which was stratified by HbA_1c_ (hazard ratio 2·0, 95% CI 0·5–8·0; p=0·33). For the sensitivity analysis of potential differential variability between the light mask and sham mask over time in the zone of maximum baseline retinal thickness, which was done in 246 patients, the difference between treatment groups was not significant from baseline to 24 months (p=0·38). A sensitivity analysis of the primary outcome in patients who had OCT measurements taken with Spectralis (Heidelberg Engineering) at baseline showed no difference between treatment groups (appendix p 12).

In the secondary analyses of retinal thickness and volume, no significant differences were noted between the light mask and sham mask in change from baseline in central subfield thickness, total thickness of central and parafoveal zones, total retinal thickness measured over all nine zones, and total macular volume at 12 months and 24 months (appendix pp 13, 14). Analysis of secondary morphological outcomes showed that significantly more patients assigned the light mask had resolution of diffuse diabetic macular oedema at 12 months (difference between groups in change from baseline, −13%, 95% CI −23 to −2; p=0·0246), but this effect was lost at 24 months (2%, −10 to 14; p=0·75). Foveal cysts were somewhat reduced at 12 months (−12%, 95% CI −25 to 0·1; p=0·052) and 24 months (−14%, −27 to 0·3; p=0·054) in patients assigned the light mask compared with those allocated the sham mask. Changes in visible cysts in the inner ETDRS zones did not differ between treatment groups but the proportion of patients with visible cysts in the outer ETDRS zones was reduced significantly more in patients assigned the light mask compared with those allocated the sham mask (appendix pp 15, 16).

The adjusted difference in best-corrected visual acuity between the light mask and sham mask was also not significant (at 12 months, −0·07 ETDRS letters, 95% CI −1·38 to 1·23; p=0·91; at 24 months, 0·13 ETDRS letters, −1·45 to 1·71; p=0·87; appendix pp 13, 14). The proportion of patients showing progression of retinopathy was low, and no difference was recorded between treatment groups at 12 months and 24 months (appendix p 17). With respect to sleep disturbances, ESS scores and PIRS-20 scores did not differ between treatment groups (appendix pp 13, 14).

The success of concealing the treatment allocation from primary assessors (OCT technicians and optometrists) was assessed with a guess form. In line with chance, OCT technicians guessed the allocation correctly for 137 (55%) of 248 patients and optometrists for 129 (52%) of 246 patients. The response was based on random choice for 180 (73%) OCT technicians and 231 (94%) optometrists, and 68 (27%) and 15 (4%), respectively, made an educated guess based on a clinical response or adverse event.

58 serious adverse events were recorded, of which 32 were reported in patients assigned the light mask and 26 were noted in those allocated the sham mask; none were related to the active intervention (appendix p 18). 340 adverse events not related to the intervention were reported, of which 172 were noted in patients assigned the light mask and 168 were in those assigned the sham mask (appendix p 19). 72 adverse events were reported as related to the assigned treatment, which included 50 in patients allocated the light mask and 22 in those assigned the sham mask (table 3). The most frequent adverse events related to the assigned treatment were discomfort on the eyes (14 with the light mask *vs* seven with the sham mask), painful, sticky, or watery eyes (14 *vs* six), and sleep disturbance (seven *vs* one).

**Table 3:** Adverse events related to intervention

	**Sham mask (n=153)**	**Light mask (n=155)**
**Eyes**		
Corneal abrasion, corneal ulcer	3	0
Mask causing pressure on eyes, pain on eyes, uncomfortable masks	7	14
Sore eyebrows, sore eyelids	0	2
Subconjunctival haemorrhage	0	1
Vision deterioration, disturbance	1	2
Watery eyes, sore eyes, sticky eyes, painful eyes, conjunctivitis	6	14
**Neurological**		
Headache, severe persistent headache	0	2
**Psychiatric**		
Insomnia	0	1
**Musculoskeletal**		
Left-sided neck and skull pain	0	1
**Dermatological**		
Scratched face on two occasions getting mask off during sleep	1	0
Sore skin, small lump on side of right eye	0	1
Pod moving around in mask when turns in bed	0	1
Wart	0	1
**Other**		
Mask slipping off head	3	3
Sleep disturbance, bad dreams	1	7

## Discussion

The CLEOPATRA trial is the first phase 3 randomised controlled trial to evaluate a light mask as an intervention to treat and prevent non-central diabetic macular oedema in a multicentre setting. Our results show that the light mask as offered in this study is not an effective option in the treatment or prevention of progression of non-central diabetic macular oedema. Although objective assessment of the reduction of maximum retinal thickness was our primary outcome, we have made our conclusion based on the primary outcome, per-protocol secondary analysis, and five prespecified sensitivity analyses of the primary outcome, and none of these analyses showed any therapeutic benefit of wearing these light masks. Moreover, because of the dynamic nature of diabetic macular oedema, we considered several secondary outcomes, including reduction in total retinal thickness, macular volume, progression of central subfield thickness to 300 μm or more, and the proportions of patients requiring treatment for new onset centre-involving diabetic macular oedema and of those treated with standard therapy during the trial due to worsening of diabetic macular oedema. None of the changes in these variables was significant between treatment groups, substantiating the results of the primary outcome. Furthermore, no treatment effect was noted in severity of diabetic retinopathy with these light masks. However, the light masks did significantly reduce diffuse diabetic macular oedema and visible cysts in outer ETDRS zones at 12 months, but this effect did not translate to a significant change in retinal thickness and the effect was not sustained at 24 months, suggesting that any positive morphological effects of these light masks on diabetic macular oedema is transient and minimal.

We expected compliance with light masks to be an issue based on findings of the phase 2 study^10^ and because non-centre-involving diabetic macular oedema is asymptomatic. Therefore, we made several efforts to tackle compliance-related issues in this study. First, we calculated the sample size with a 20% attrition rate, which is higher than most ophthalmic trials. Furthermore, we allowed for OCT measurements from clinic appointments to be used when patients attended the clinic visit and not a clinical trial visit appointment. We had also carefully considered the effect of non-compliance on the potential therapeutic effect of the light masks by incorporating a predefined CACE analysis for non-compliance at three levels—70%, 60%, and 50%. Non-compliance was noted as early as 4 months into the trial and across all three definitions of compliance.

Compliance with use of light masks has varied between studies and can be partly explained by the differences in definitions used in determining compliance levels.^10^, ^20^, ^21^, ^22^, ^23^ However, this study is the first randomised trial evaluating the use of a light mask during sleep at night over 24 months and shows that compliance reduces over time in keeping with the adherence patterns of self-management strategies in asymptomatic diabetes.^24^ Decline in adherence is rapid after the first 6 months of therapy in chronic diseases.^25^ The compliance levels observed in our study are in keeping with the WHO report^25^ that shows that the mean adherence to long-term therapy in patients with chronic diseases is approximately 50%. Therefore, further studies should include additional interventions to increase patient engagement in wearing the light masks to evaluate whether this intervention is sustainable over the lifetime of their diabetic eye disease.

Both treatment groups showed a gradual mean reduction in the zone of maximum retinal thickness over 24 months. The reduction is within the SD that we used for the sample size calculation. The event rate of progression to centre-involving diabetic macular oedema was also similar to findings of previous reports.^2^, ^5^

The main strength of our study was that we ensured that the primary outcome was corroborated by a predefined sensitivity analysis and secondary analysis to reduce potential systemic biases in this dynamic condition. Other strengths included clear definition of objective endpoints, publication of the protocol, substantial public and patient involvement throughout the study, and strict assessment-assured high-quality data and preplanned analysis for expected non-compliance. The baseline characteristics of the trial population were typical for the intended patient population. The representative multiethnic patient population, together with the multicentre trial design, permit wide generalisability of our results.

Nevertheless, some limitations should be considered when interpreting the results. First, the study only shows that offering the light mask as per this study protocol to suppress rod function is not an effective option to treat non-central diabetic macular oedema. It is possible that the retinal illumination achieved with these devices did not reduce the dark current sufficiently to alter the hypoxic state. Therefore, it is worth evaluating other techniques of rod suppression in diabetic retinopathy and diabetic macular oedema since there is a growing body of scientific evidence that supports the role of photoreceptors in retinal vascular permeability and angiogenesis.^26^, ^27^ Other clinical trials of light masks to prevent dark adaptation in diabetic retinopathy are ongoing. The Lahey Light II trial^20^ (LCID Study Number 2015-020) is evaluating a modified 520 μm LED light mask to prevent dark adaptation in refractory diabetic macular oedema. Two other clinical trials (ISRCTN82148651 and NCT02207712) are ongoing for age-related macular degeneration. However, it is important that studies with long-term follow-up are conducted in these chronic conditions to provide further insight into this intervention. Second, it could be argued that hypoxia might not be a contributing factor in early diabetic macular oedema and that patients with early signs of this disorder might not be the ideal target population. However, oxygen therapy has been shown to ameliorate early diabetic macular oedema, reinforcing the role of hypoxia in this condition.^9^, ^28^ A third limitation is that we defined non-centre-involving diabetic macular oedema as a zone of retinal thickness above 320 μm. Although the normative data of some zones on Spectralis OCT could in fact be above 320 μm, we only included eyes with clinical evidence of diabetic macular oedema causing the retinal thickness to be greater than 320 μm. Therefore, we believe that our patient population is representative of early non-central diabetic macular oedema. In our study, there was no discernible treatment effect in favour of the light mask at 4 months and 8 months when compliance was highest; in fact, any effect was in the opposite direction, which was maintained at 12 months, suggesting that low compliance did not contribute significantly to the overall study result.

In conclusion, the light mask as offered in this study is not an effective intervention to prevent or treat patients with non-centre-involving diabetic macular oedema. Future trials should aim to identify better ways of rod suppression to assess the role of rods in diabetic macular oedema and diabetic retinopathy.
