# Supplementary material for: Clinical efficacy and safety of a light mask for prevention of dark adaptation in treating and preventing progression of early diabetic macular oedema at 24 months (CLEOPATRA): a multicentre, phase 3, randomised controlled trial
Source: Lancet Diabetes Endocrinol. 2018 May;6(5):382–91. doi: 10.1016/S2213-8587(18)30036-6 (PMC5908782; doi:10.1016/S2213-8587(18)30036-6)
Supplement: Supplementary appendix [file mmc1.pdf]

THE LANCET
Diabetes & Endocrinology

Supplementary appendix

This appendix formed part of the original submission and has been peer reviewed. We post it as supplied by the authors.

Supplement to: Sivaprasad S, Vasconcelos J C, Prevost A T. Clinical efficacy and safety of a light mask for prevention of dark adaptation in treating and preventing progression of early diabetic macular oedema at 24 months (CLEOPATRA): a multicentre, phase 3, randomised controlled trial. *Lancet Diabetes Endocrinol* 2018; published online Mar 5. http://dx.doi.org/10.1016/S2213-8587(18)30036-6.

Table S1: Clinical assessment schedule

	**Screening /Baseline**	**Week 1**	**Month 4**	**Month 8**	**Month 12**	**Month 16**	**Month 20**	**Month 24 End of Trial**	**Withdrawal**
**Study week/month**	Visit 1	Visit 2	Visit 3	Visit 4	Visit 5	Visit 6	Visit 7	Visit 8	
Informed consent	x								
Eligibility form	x								
Medical history	x								
Concomitant medications	x		x	x	x	x	x	x	x
Ocular examination	x		x	x	x	x	x	x	x
HbA_{1c} and BP	x				x			x	x
Pittsburgh Insomnia Rating (PIRS 20) Score and Epworth Sleepiness Scale (ESS)	x	x			x			x	x
BCVA*	x*		x	x	x*	x	x	x*	x*
VA repeated with new refraction	x								
OCT –macular thickness protocol	x		x	x	x	x	x	x	x
Repeat OCT- macular thickness protocol					x			x	x
Retinal colour photographs: 3-fields	x				x			x	x
Mask compliance		x	x	x	x	x	x	x	x
Adverse events form		x	x	x	x	x	x	x	x
Randomisation form	x								
Withdrawal form									x
Assessor outcome guess								x	x

BCVA=best corrected visual acuity; OCT=optical coherence tomography; HbA_{1c}=glycated haemoglobin; BP=blood pressure; VA=visual acuity. *Refraction at screening, 12 and 24 months.

Table S2: ETDRS diabetic retinopathy severity score levels

ETDRS level	ETDRS severity	ETDRS definition
10	No retinopathy	Diabetic retinopathy absent
20	Very mild NPDR	Microaneurysms only
35	Mild NPDR	Hard exudates, cotton-wool spots, and/or mild retinal haemorrhages
43	Moderate NPDR	43A:retinal haemorrhages moderate (>photograph 1A) in 4 quadrant or severe (≥ photograph 2A) in 1 quadrant<br>43B:mild IRMA (<photograph 8A) in 1 to 3 quadrants
47	Moderate NPDR	47A:both level 43 characteristics<br>47B:mild IRMA in 4 quadrants<br>47C:severe retinal haemorrhage in two to three quadrants<br>47D:venous beading in one quadrant"
53	Severe NPDR	53A:≥2 level 47 characteristics<br>53B:severe retinal haemorrhages in 4<br>53C:moderate to severe IRMA (≥ photograph 8A) in at least 1 quadrant<br>53D:venous beading in at least 2 quadrants<br>53E: ≥2 level 53A-D characteristics
61	Mild PDR	NVE <0·5 disk area in 1 or more quadrants
65	Moderate PDR	65A:NVE≥0·5 disk area in 1 or more quadrants<br>65B:NVD<photograph 10A (0·25-0·33 disk area)
71 and 75	High-risk PDR	NVD ≥ photograph 10A, or NVD < photograph 10A or NVE ≥ 0·5 disk area plus VH or PRH, or VH or PRH obscuring ≥ 1 disk area

81 and 85	Advanced PDR	Fundus partially obscured by VH and either new vessels ungradable or retina detached at the centre of the macula
90	Ungradable	Poor image quality

ETDRS= early treatment diabetic retinopathy study; NPDR=Non-proliferative diabetic retinopathy; PDR=Proliferative Diabetic Retinopathy; IRMA=Intraretinal Microvascular Abnormalities; NVE=neovascularisation elsewhere; NVD=noevascularisation disc; VH=vitreous haemorrhage; PRH=pre-retinal haemorrhage.

Table S3: Differences in baseline characteristics between those with and without primary outcome data at 24 months

	Without 24 month data (N=62)	With 24 month data (N=246)	*p*-value
**Mean (SD) age**	57·2 (12·9)	57·5 (11·0)	0·87[1]
**Women % (n)**	40% (25)	36% (89)	0·55[2]
**Ethnicity**			
White	76% (47)	60% (147)	
Black	16% (10)	19% (46)	
Asian	8% (5)	19% (47)	0·07[3]
Other	0% (0)	2% (6)	
**Smoker % (n)**	7% (4)	8% (19)	0·73[2]
**Mean (SD) baseline maximal zone retinal thickness**	348·1 (22·4)	347·2 (23·2)	0·77[1]
**Mean (SD) baseline BCVA**	83·8 (7·6)	84·4 (7·3)	0·59[1]
**Site % (n)**			
Bristol Eye Hospital	7% (4)	3% (8)	
Birmingham Heartlands Hospital	3% (2)	4% (9)	
Sandwell & West Birmingham Hospitals NHS Trust	3% (2)	2% (4)	
Frimley Park Hospital	23% (14)	7% (18)	
Hillingdon Hospital	8% (5)	20% (49)	
King's College Hospital	8% (5)	12% (30)	0·0143[3]
Moorfields Eye Hospital	10% (6)	18% (45)	
Central Middlesex Hospital	3% (2)	1% (2)	
Maidstone & Tunbridge Wells Hospital	3% (2)	4% (10)	
Princess Alexandra Hospital, Harlow	3% (2)	4% (9)	
The Royal Wolverhampton NHS Trust	5% (3)	7% (18)	
Brighton & Sussex University Hospitals NHS Trust	0% (0)	1% (3)	
Sunderland Eye Infirmary	11% (7)	7% (18)	
Stoke Mandeville Hospital	2% (1)	4% (9)	
William Harvey Hospital Kent	11% (7)	6% (14)	

BCVA=best corrected visual acuity. [1]T-test, [2]Chi-squared test, [3]Fisher's Exact test. For site, *p*-value was computed using Monte Carlo simulation.

Table S4: **Number of participants followed up at each timepoint**

Timepoint	No. participants followed up			No. participants followed up with primary outcome[1]		
	**Non-light mask**	**Light mask**	**Total**	**Non-light mask**	**Light mask**	**Total**
Baseline	153	155	308	153	155	308
4 m	121	135	256	119	132	251
8 m	112	113	225	113	113	226
12 m	121	130	251	121	130	251
16 m	102	111	213	103	111	214
20 m	102	100	202	104	100	204
**24 m**	**115**	**122**	**237**	**119**	**127**	**246**

[1]This includes participants who had clinical optical coherence tomography data

Table S5: Comparison of the primary outcome between arms at all time-points

	4 months	8 months	12 months	16 months	20 months	24 months
**Mean adjusted difference in retinal thickness (**μm)	4·64	2·35	1·73	-0·27	2·46	-0·65
**95% CI**	(-1·02, 10·3)	(-3·28, 7·99)	(-5·31, 8·77)	(-6·67, 6·13)	(-3·95, 8·87)	(-6·90, 5·59)

Table S6: Number of participants requiring treatment (steroids, anti-VEGF or laser) at each follow-up visit (per protocol sample)

**Month visit:**	**Number of participants requiring treatment**			**Number of participants with primary outcome at each follow-up visit after removing those requiring treatment**		
	**Non-light mask**	**Light mask**	**Total**	**Non-light mask N=153**	**Light mask N=155**	**Total N=308**
4 months	7	4	11	112	128	240
8 months	6	3	9	102	106	208
12 months	9	5	14	102	120	222
16 months	6	4	10	81	97	178
20 months	1	6	7	80	82	162
24 months	4	1	5	90	105	195

VEGF=vascular endothelial growth factor.

Table S7: Per protocol analysis of the primary outcome compared between arms at 12 and 24 months

Primary Outcome: OCT	Mean (SD); N		Change from baseline mean (SE)		Adjusted difference between arms (95% CI)	*p*-value
	Non-lightmask	Lightmask	Non-lightmask	Lightmask		
**Baseline**	348·8 (24·3); 153	347·7 (31·2); 155	-	-	-	-
**12-months**	333·4 (26·6); 102	339 (18·6); 120	-13·1 (2·9)	-5·3 (1·8)	4·50 (-0·72, 9·73)	0·09
**24-months**	330·5 (21·6); 90	335·9 (21·9); 105	-14·6 (2·8)	-8·5 (2·1)	3·23 (-2·11, 8·58)	0·23

Note: 266 patients were included in the LME models (at 12 and 24 months)· The reason for including 11 fewer participants in PP than ITT is because these patients "took" some treatment at the 4 month visit and therefore do not provide any follow-up measurement to be accounted for in the model. OCT=optical coherence tomography.

Table S8: Median (IQR) level of compliance from baseline over time and percentage (number) of participants achieving different levels of compliance

	4 months	8 months	12 months	16 months	20 months	24 months
**Median**	39·5	32·0	25·7	25·1	20·5	19·5
**IQR**	9·8 - 78·2	5·4 - 68·6	3·8 - 64	2·8 - 60·7	2·3 - 55·6	1·9 - 51·6
**Levels of compliance:**						
**70%**	31% (48/155)	23% (36/155)	21% (32/155)	19% (30/155)	19% (29/155)	16% (24/155)
**60%**	38% (59/155)	32% (49/155)	28% (44/155)	26% (40/155)	23% (35/155)	21% (33/155)
**50%**	45% (69/155)	39% (61/155)	36% (56/155)	33% (51/155)	30% (47/155)	26% (40/155)

Table S9: Sensitivity analysis for missing data

**Three scenarios reflecting whether departures from the missing at random assumption apply within:**	**Retinal thickness range over which the mean of the "unobserved outcome data" might depart from the mean of the "observed outcome data"**	
	**-20µm**	**+20µm**
1: the intervention arm only (light masks)	-4·3 (-10·5, 2)	3·0 (-3·3, 9·2)
2: the control arm only (non-light masks)	-5·1 (-11·3, 1·1)	3·8 (-2·5, 10·0)
3: both arms equally and in the same direction	0·2 (-6·1, 6·4)	-1·5 (-7·7, 4·8)

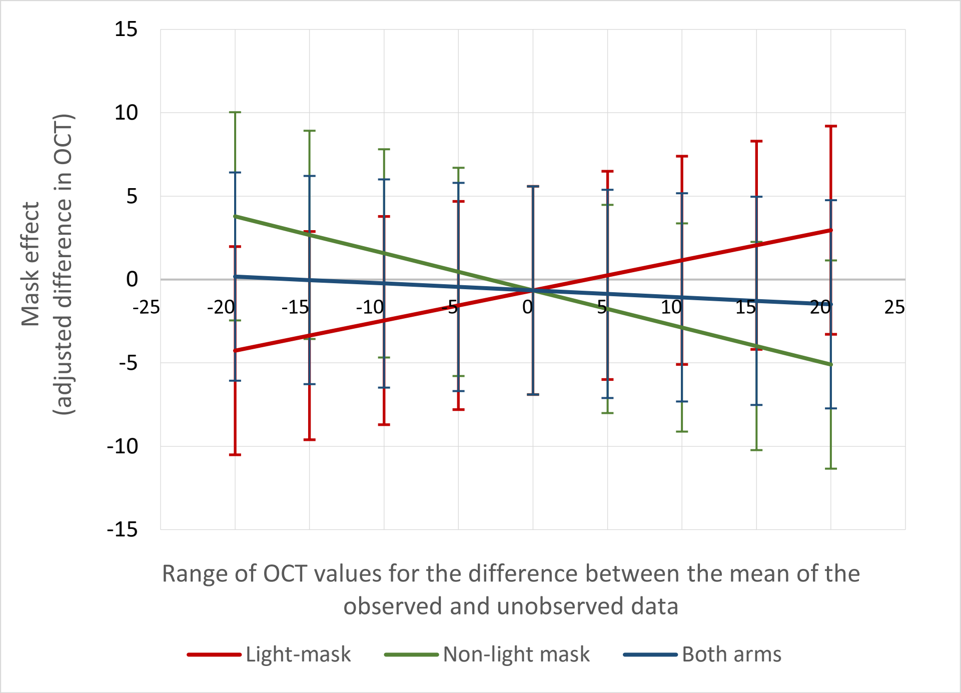

Figure S1: Sensitivity analysis to the missing at random assumption made in primary outcome analysis

OCT=optical coherence tomography.

Table S10: Sensitivity analysis including only those participants who had the Spectralis OCT (Heidelberg Engineering, Heidelberg, Germany) measurements at baseline

Primary Outcome: OCT	Mean (SD); N		Change from Baseline Mean (SE)		Adjusted difference between arms (95% CI)	*p*-value
	Non-light mask	Light mask	Non-light mask	Light mask		
**Baseline**	353·8 (26·0); 92	349·7 (20·6); 91	-	-	-	-
**At 12-months**	344·2 (35·2); 73	345·1 (32·7); 74	-9·4 (4·1)	-5·0 (3·8)	0·15 (-9·83, 10·13)	0·98
**At 24-months**	340·3 (28·4);70	339·8 (23·3); 72	-13·9 (4·0)	-9·3 (2·9)	-0·22 (-8·36, 7·92)	0·96

Note: Included in the linear mixed effects (LME) models at 12 and 24months are 161 participants. OCT=optical coherence tomography.

Table S11: Change in retinal thickness outcomes, macular volume, visual acuity, sleep disturbance measures and HbA_{1c}

	Mean (SD); N		Change from baseline Mean (SE)		Adjusted difference between arms (95% CI) (Light– non-light mask)	*p*-value
	Non-light mask	Light mask	Non-light mask	Light mask		
**RT in the central zone**						
Baseline	271·9 (18·9); 153	269·3 (18·9); 155	-	-	-	-
12-months	273·8 (34·3); 121	274·5 (36·7); 132	2·9 (2·6)	4·9 (2·8)	1·59 (-5·80, 8·98)	0·67
24-months	275·1 (42·3); 119	274·6 (37·4); 127	2·7 (3·4)	5·2 (3·2)	2·74 (-6·39, 11·88)	0·55
**RT in the central and parafoveal zones**						
Baseline	1604 (83); 153	1591 (82); 155	-	-	-	-
12-months	1599 (127); 121	1600 (121); 132	-1·1 (7·9)	7·8 (7·9)	5·36 (-16·17, 26·90)	0·62
24-months	1597 (137); 119	1589 (124); 127	-6·1 (9·9)	-0·2 (8·4)	4·72 (-20·3, 29·74)	0·71
**RT in over the 9 zones**						
Baseline	2789 (157); 153	2764 (142); 155	-	-	-	-
12-months	2781 (213); 121	2774 (187); 132	2·0 (11·9)	7·5 (11·3)	-0·26 (-31·68, 31·15)	0·99
24-months	2772 (221); 119	2756 (196); 126	-11·2 (14·7)	-6·9 (12·4)	1·52 (-34·78, 37·83)	0·93
**Macular Volume**						
Baseline	8·9 (0·9); 153	8·8 (0·8); 155	-	-	-	-
12-months	8·9 (0·9); 122	8·8 (0·9); 132	0·0 (0·0)	-0·1 (0·0)	-0·06 (-0·19, 0·06)	0·34
24-months	8·9 (1·2); 121	8·9 (0·9); 126	0·0 (0·1)	0·0 (0·0)	-0·01 (-0·15, 0·14)	0·92
**BCVA refracted**						
Baseline	84·3 (7·3); 153	84·3 (7·4); 155	-	-	-	-
12-months	83·5 (9·1); 120	83·6 (8·2); 131	-0·6 (0·5)	-0·7 (0·4)	-0·07 (-1·38, 1·23)	0·91
24-months	82·4 (10·1); 115	83·0 (8·8); 122	-1·8 (0·7)	-1·6 (0·5)	0·13 (-1·45, 1·71)	0·87
**Daytime sleepiness (ESS)**						
Baseline	6·2 (4·0); 143	5·7 (3·7); 148				
12-months	6·1 (4·2); 112	5·5 (4·1); 120	-0·3 (0·4); 109	0·2 (0·3); 116	-0·11 (-1·05, 0·83)	0·82
24-months	5·8 (4·7); 107	5·7 (4·4); 118	-0·3 (0·4); 102	0·4 (0·3); 112	0·51 (-0·42, 1·43)	0·28
**Insomnia (PIRS 20)**						
Baseline	13·6 (10·4); 146	12·7 (10·3); 152				
12-months	13·6 (10·1); 114	14·5 (11·7); 121	-0·2 (0·8); 110	2·0 (0·9); 119	1·56 (-0·62, 3·74)	0·16
24-months	13·2 (9·5); 111	14·2 (12·4); 116	-0·4 (1·0); 106	1·4 (1·0); 114	1·41 (-1·02, 3·84)	0·25

**HbA_{1c}**						
Baseline	67·2 (18); 153	68·5 (19·4); 155				
12-months	70·9 (20·1); 119	68·4 (19·9); 130	3·1 (1·6)	-0·1 (1·0)	-2·73 (-6·31, 0·85)	0·13
24-months	68·1 (16·4); 111	66·9 (18·6); 121	0·3 (1·4)	-1·1 (1·3)	-1·55 (-4·91, 1·80)	0·36

BVCA-best-corrected visual acuity; RT-retinal thickness; HbA_{1c}=glycated haemoglobin; PIRS 20=Pittsburgh Insomnia Rating Score and ESS=Epworth Sleepiness Scale

Table S12: Changes in morphological characteristics of macular thickening on OCT at 12 and 24 months

	Non-light mask % (n/N)	Light mask % (n/N)	Difference between arms in change from baseline 95% CI	*p*-value
**Hyporeflectivity of outer nuclear layer without cysts (diffuse)**				
Baseline	18% (27/153)	19% (30/155)		
12 months	21% (26/121)	11% (14/132)		
Change from baseline to 12m	5% (6/121)	-8% (-10/132)	-13% (-23%, -2%)	0·0246
24 months	12% (14/118)	13% (16/123)		
Change from baseline to 24m	-6% (-7/118)	-4% (-5/123)	2% (-10%, 14%)	0·75
**Visible cysts in foveal area**				
Baseline	16% (24/153)	24% (37/155)		
12 months	26% (32/121)	23% (30/132)		
Change from baseline to 12m	10% (12/121)	-2% (-3/132)	-12% (-25%, 0%)	0·052
24 months	29% (34/118)	25% (31/123)		
Change from baseline to 24m	13% (15/118)	-1% (-1/123)	-14% (-27%, 0%)	0·054
**Visible cysts in inner ETDRS zones (<3 mm)**				
Baseline	47% (72/153)	44% (68/155)		
12 months	38% (46/120)	40% (53/132)		
Change from baseline to 12m	-12% (-14/120)	-2% (-3/132)	9% (-4%, 23%)	0·18
24 months	39% (46/118)	33% (40/122)		
Change from baseline to 24m	-10% (-12/118)	-11% (-14/122)	-1% (-16%, 13%)	0·86
**Visible cysts in outer ETDRS zones (3-6mm)**				
Baseline	38% (58/152)	41% (64/155)		
12 months	38% (46/120)	26% (34/132)		
Change from baseline to 12m	0% (0/120)	-14% (-18/132)	-14% (-27%, -1%)	0·0398
24 months	37% (44/118)	33% (41/123)		
Change from baseline to 24m	-1% (-1/118)	-5% (-6/123)	-4% (-17%, 9%)	0·55
**VMT**				
Baseline	0% (0/153)	2% (3/155)		
12 months	0% (0/121)	2% (3/132)		
Change from baseline to 12m	0% (0/121)	0% (0/132)	0% (-3%, 3%)	1·00
24 months	3% (4/118)	1% (1/123)		
Change from baseline to 24m	3% (4/118)	-2% (-2/123)	-5% (-9%, -1%)	0·0130
**Subretinal fluid**				
Baseline	0% (0/153)	1% (1/155)		
12 months	1% (1/121)	0% (0/132)		
Change from baseline to 12m	1% (1/121)	-1% (-1/132)	-2% (-4%, 1%)	0·16

24 months	0% (0/118)	2% (2/123)		
Change from baseline to 24m	0% (0/118)	1% (1/123)	1% (-2%, 4%)	0·56
**Epiretinal membrane**				
Baseline	1% (2/153)	2% (3/155)		
12 months	2% (3/121)	3% (4/132)		
Change from baseline to 12m	1% (1/121)	1% (1/132)	0% (-4%, 4%)	0·97
24 months	7% (8/118)	4% (5/123)		
Change from baseline to 24m	5% (6/118)	2% (2/123)	-3% (-10%, 3%)	0·29

ETDRS=early treatment diabetic retinopathy study; OCT=optical coherence tomography; VMT=vitreomacular traction.

Table S13: Proportion of participants that show progression to retinopathy (ETDRS severity levels)

	Non-light mask % (n/N)	Light mask % (n/N)	Difference in proportions (95%CI)
12 months (≥2 ETDRS level improvement)	1% (1/84)	1% (1/87)	-0.04% (-5.4% to 5.4%)
24 months (≥2 ETDRS level improvement)	3% (3/96)	3% (3/99)	-0.09% (-6.5% to 6.5%)

ETDRS= early treatment diabetic retinopathy study

Table S14: Number of serious adverse events by body system and arm

	Non-light mask	Light mask	Total
Cardiovascular	8	4	12
Respiratory	3	1	4
Hepatic	1	1	2
Gastro-intestinal	2	6	8
Genito-urinary	2	0	2
Endocrine	3	2	5
Musculoskeletal	3	6	9
Neurological	2	6	8
Psychiatric	0	1	1
Dermatological	2	1	3
Other	0	4	4
**Total**	**26**	**32**	**58**

Table S15: Adverse events not related to intervention

Body System	Non-light mask	Light mask	Total
Eyes	51	40	91
Cardiovascular	5	5	10
Respiratory	23	13	36
Gastrointestinal	8	17	25
Genito-urinary	12	9	21
Endocrine	6	2	8
Haematological	4	2	6
Musculoskeletal	16	24	40
Neurological	5	16	21
Psychiatric	3	5	8
Immunological	3	2	5
Dermatological	11	6	17
Allergies	2	1	3
Ear, nose, throat	13	14	27
Other	6	16	22
**Total**	**168**	**172**	**340**
